# Comparison of Out-of-Hospital Cardiac Arrests and Fatalities in the Metro Detroit Area During the COVID-19 Pandemic With Previous-Year Events

**DOI:** 10.1001/jamanetworkopen.2020.32331

**Published:** 2021-01-06

**Authors:** Adrienne V. Nickles, Adam Oostema, Justin Allen, Suzanne L. O’Brien, Stacie L. Demel, Mathew J. Reeves

**Affiliations:** 1 Lifecourse Epidemiology and Genomics Division, Michigan Department of Health and Human Services, Lansing; 2 Department of Emergency Medicine, College of Human Medicine, Michigan State University, East Lansing; 3 Division of Chronic Disease and Injury Control, Michigan Department of Health and Human Services, Lansing; 4 Department of Neurology, University of Cincinnati, Cincinnati, Ohio; 5 Department of Epidemiology, College of Human Medicine, Michigan State University, East Lansing

## Abstract

This cross-sectional study compares trends in out-of-hospital cardiac arrests and fatalities in the Detroit area during the COVID-19 pandemic with year-earlier events for the same period.

## Introduction

It is difficult to overstate the impact of the coronavirus disease 2019 (COVID-19) pandemic. As of November 30, 2020, there were more than 63 million documented infections and approximately 1.5 million deaths worldwide.^1^ COVID-19 outbreaks have been especially acute in certain regions in the United States. In Michigan, 360 449 confirmed infections with severe acute respiratory syndrome coronavirus 2 (SARS-CoV-2), the virus that causes COVID-19, have occurred since the first documented cases on March 10, 2020.^2^ Nearly 33% of these cases occurred in the metropolitan Detroit area.^2^ Approximately 60% of these cases occurred in the metropolitan Detroit area (Macomb, Oakland, and Wayne counties), an area with a combined population of 3.9 million. Reports from Italy,^3^ France,^4^ and New York^5^ have documented marked increases in out-of-hospital cardiac arrests (OHCAs) attended by emergency medical services (EMS) in areas with significant COVID-19 burdens. We examined whether a similar phenomenon occurred in the metropolitan Detroit area during the peak of the outbreak by using data from the Michigan EMS Information System.

## Methods

Nontraumatic OHCA calls in Macomb, Oakland, and Wayne counties between January 1 and May 31 of both 2019 and 2020 were identified from the Michigan EMS Information System. Basic Life Support and Medical First Response records were excluded to eliminate duplicate counting of events.

Weekly per capita OHCA incidence rates (events per 100 000 population) were calculated for each year. We also calculated weekly per capita SARS-CoV-2 incidence rates per 100 000 population for the 3 counties using the official count of confirmed cases from the Michigan Department of Health and Human Services.^2^ We used χ^2^ tests to compare demographic and clinical characteristics of OHCA events after Michigan’s stay-at-home order (March 23 through May 31, 2020) with the same period in 2019. This cross-sectional study followed the Strengthening the Reporting of Observational Studies in Epidemiology (STROBE) reporting guidelines. Because this study was a public health surveillance activity authorized by a public health authority, it did not require institutional review board approval per 45 CFR 46.102(l)(2). Patient informed consent for this study was waived per the State of Michigan Public Health Code (Michigan Public Health Code Act, PA 368 of 1978 Sec. 1101). Two-sided *P* < .05 was used to test statistical significance. All analyses were performed using SAS statistical software, version 9.4 (SAS Institute Inc).

## Results

Between March 23 and May 31, 2019, there were 1162 OHCA calls identified from the Michigan EMS Information System. Of these, 451 OHCAs (38.8%) occurred in individuals aged 65 to 84 years, 662 (57.0%) occurred in men, and 626 (53.9%) occurred in White individuals (Table). During the same period in 2020, 1854 OHCA calls were identified. Of these, 735 OHCAs (39.6%) occurred in individuals aged 65 to 84 years, 1084 (48.5%) occurred in men, and 867 (46.8%) occurred in White individuals.

**Table.** Comparison of Demographic and Clinical Characteristics of Calls for Out-of-Hospital Cardiac Arrests Between March 23 and May 31 in 2019 and 2020

Characteristic	No. (%)		*P* value
	2019 (n = 1162)	2020 (n = 1854)	
Age category, y			
0-49	230 (19.8)	301 (16.2)	.01
50-64	304 (26.2)	474 (25.6)	
65-84	451 (38.8)	735 (39.6)	
≥85	171 (14.7)	341 (18.4)	
Male^a^	662 (57.0)	1083 (58.5)	.46
Race/ethnicity			
Black	353 (30.4)	724 (39.1)	<.001
White	626 (53.9)	867 (46.8)	
Missing	162 (13.9)	232 (12.5)	
Other^b^	21 (1.8)	31 (1.7)	
Location			
Home or private residence	800 (68.8)	1191 (64.2)	.03
SNF, AL, or institutional residence	219 (18.8)	407 (22.0)	
Other or not recorded	143 (12.3)	256 (13.8)	
Presumed cause			
Cardiac	930 (80)	1502 (81)	.08
Respiratory or asphyxia	71 (6.1)	141 (7.6)	
Drug overdose	59 (5.1)	68 (3.7)	
Other or not recorded	102 (8.8)	143 (7.7)	
Intervention			
CPR	580 (49.9)	847 (45.7)	.06
Advanced airway placement	529 (45.5)	397 (21.4)	<.001
Death on scene	619 (53.3)	1400 (75.5)	<.001
No resuscitation	267 (23.0)	544 (29.3)	<.001

Abbreviations: AL, assisted living; CPR, cardiopulmonary resuscitation; SNF, skilled nursing facility.

^a^ Sex data were unknown for 3 cases.

^b^ The other race/ethnicity category includes Asian, American Indian/Alaska Native, Native Hawaiian or other Pacific Islander, Hispanic or Latino, or the selection of “other race” by the emergency medical services provider.

The Figure shows the incidence of OHCA events per capita from January through May of 2019 and 2020 along with the incidence of confirmed COVID-19 cases in 2020. Beginning in early March 2020 (week 12), a marked increase in the incidence of OHCAs was observed compared with the previous year. The spike in OHCAs lags slightly behind but closely mirrors the epidemic curve of COVID-19 (which represents the date of symptom onset for confirmed cases) during the same period.

**Figure.**
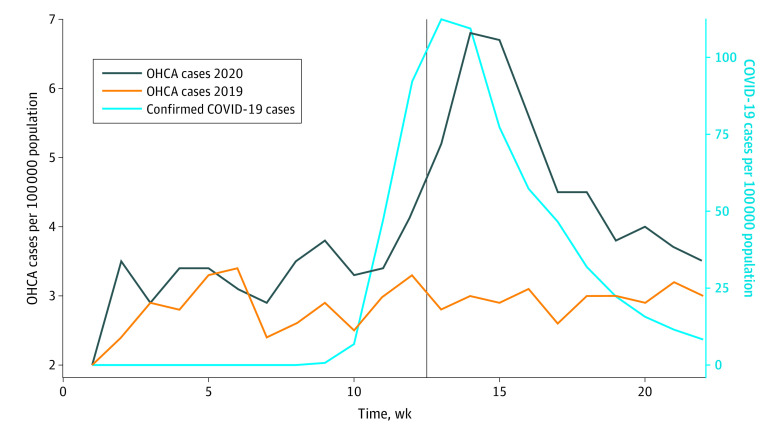
Weekly per Capita Rates of Out-of-Hospital Cardiac Arrest (OHCA) Calls Between January 1 and May 31 in 2019 and 2020 and Weekly per Capita Rates of COVID-19 Diagnoses During 2020

The 1854 OHCA calls during the pandemic period studied (March 23 through May 31, 2020) represent a 60% increase compared with 1162 OHCA calls during the same period in 2019. The Table provides additional demographic characteristics and the presumed causes of cardiac arrest during the pandemic period compared with the same period in 2019. Out-of-hospital cardiac arrests increased across all demographic characteristics in 2020. However, the increases occurred disproportionately among elderly individuals 85 years or older (341 cases [18.4%] in 2020 vs 171 [14.7%] in 2019; *P* = .01) and Black individuals (724 cases [39.1%] in 2020 vs 353 [30.4%] in 2019; *P* < .001), consistent with data from New York City.^5^ In addition, a greater proportion of OHCA cases occurred at nursing facilities in 2020 (407 cases [22.0%] in 2020 vs 219 [18.8%] in 2019; *P* = .03). In 2020, patients with OHCAs were 53% less likely to receive an advanced airway device, such as intubation or placement of a supraglottic airway, compared with 2019 (397 patients [21.4%] in 2020 vs 529 [45.5%] in 2019; *P* < .001). The proportion of OHCA calls for patients who died in the field increased by 42% (1400 patients [75.5%] in 2020 vs 619 [53.3%] in 2019; *P* < .001).

## Discussion

Southeast Michigan experienced marked increases in both the number of OHCA calls and the prehospital fatality rates for OHCA. In 2020, OHCA disproportionately increased among older individuals, Blacks, and residents of skilled nursing facilities. This study was limited to prehospital records; definitive causes of death are not known and it is not clear from these data whether the increase arose as a direct effect of COVID-19 infection or from indirect effects of the pandemic on utilization of EMS.^6^ Further investigation is needed to characterize the phenomena underlying these associations to design interventions to mitigate the impacts of the ongoing COVID-19 pandemic.

## References

[1] Dong E, Du H, Gardner L An interactive web-based dashboard to track COVID-19 in real time. Lancet Infect Dis. 2020;20(5):533-534. doi:10.1016/S1473-3099(20)30120-1 32087114 PMC7159018

[2] Michigan Department of Health and Human Services Coronavirus cases by county by date. Public Use Dataset. Vol 2020 Michigan Dept of Health and Human Services; Published 2020. Accessed December 1, 2020. https://www.michigan.gov/coronavirus/0,9753,7-406-98163_98173---,00.html

[3] Baldi E, Sechi GM, Mare C, ; Lombardia CARe Researchers Out-of-hospital cardiac arrest during the Covid-19 outbreak in Italy. N Engl J Med. 2020;383(5):496-498. doi:10.1056/NEJMc2010418 32348640 PMC7204428

[4] Marijon E, Karam N, Jost D, Out-of-hospital cardiac arrest during the COVID-19 pandemic in Paris, France: a population-based, observational study. Lancet Public Health. 2020;5(8):e437-e443. doi:10.1016/S2468-2667(20)30117-1 32473113 PMC7255168

[5] Lai PH, Lancet EA, Weiden MD, Characteristics associated with out-of-hospital cardiac arrests and resuscitations during the novel coronavirus disease 2019 pandemic in New York City. JAMA Cardiol. 2020;5(10):1154-1163. doi:10.1001/jamacardio.2020.2488 32558876 PMC7305567

[6] Lange SJ, Ritchey MD, Goodman AB, Potential indirect effects of the COVID-19 pandemic on use of emergency departments for acute life-threatening conditions—United States, January-May 2020. MMWR Morb Mortal Wkly Rep. 2020;69(25):795-800. doi:10.15585/mmwr.mm6925e2 32584802 PMC7316316

