# Errors in Introduction, Results Section, and Table and Omitted Caption for Figure

**DOI:** 10.1001/jamanetworkopen.2020.37142

**Published:** 2021-01-27

In the Research Letter titled “Comparison of Out-of-Hospital Cardiac Arrests and Fatalities in the Metro Detroit Area During the COVID-19 Pandemic With Previous-Year Events,”^1^ published January 6, 2021, an error occurred in the Introduction, a number and its percentage in the Results section and the Table were incorrectly reported, and a caption was inadvertently omitted for the Figure. In the Introduction, the fifth and sixth sentences should have appeared as a single sentence that read as follows: “Nearly 33% of these cases occurred in the metropolitan Detroit area (Macomb, Oakland, and Wayne counties), an area with a combined population of 3.9 million.^2^” In the first paragraph of the Results section, last sentence, as well as in the Table, the number and percentage for men with out-of-hospital cardiac arrests in 2020 should have appeared as 1083 (58.4%). In addition, the caption for the Figure should have read as follows: “The vertical black line indicates the date of Michigan’s stay-at-home order.” This article has been corrected.^1^
